# Supplementary material for: MR elastography identifies regions of extracellular matrix reorganization associated with shorter survival in glioblastoma patients
Source: Neurooncol Adv. 2023 Mar 5;5(1):vdad021. doi: 10.1093/noajnl/vdad021 (PMC10102831; doi:10.1093/noajnl/vdad021)
Supplement: vdad021_suppl_Supplementary_Material [file vdad021_suppl_supplementary_material.docx]

# MR Elastography Identifies Regions of Extracellular Matrix Reorganization Associated with Shorter Survival in Glioblastoma Patients – Supplementary material

*
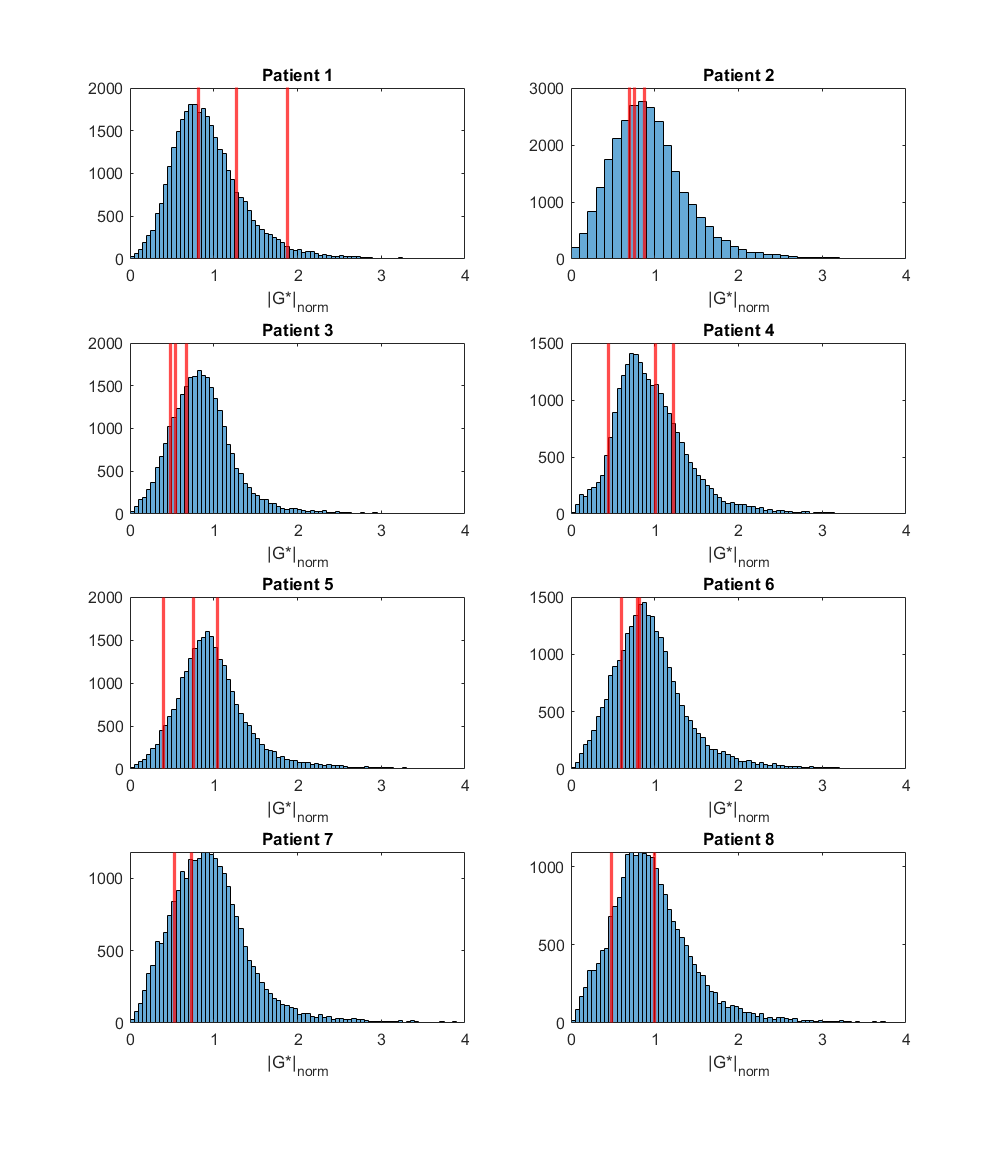
*

***Supplementary Figure 1: Distribution of |G*|_norm_*** ***for the patients analyzed using RNA sequencing.*** *Histograms show distributions of |G*|_norm_ in all voxels. Red vertical lines show the measured |G*|_norm_ for each biopsy used for RNA sequencing. The classification of biopsies as ‘stiff’ or ‘soft’ were done based on higher or lower |G*|_norm_ that the average of the 2 or 3 biopsies for that patient. As shown in this figure, for some patients this split corresponded to values below and above the most frequent value (patient 4,5 and 8). For others, all biopsies had |G*|_norm_ higher (patient 1) or lower (patient 7) than the most frequent value.*


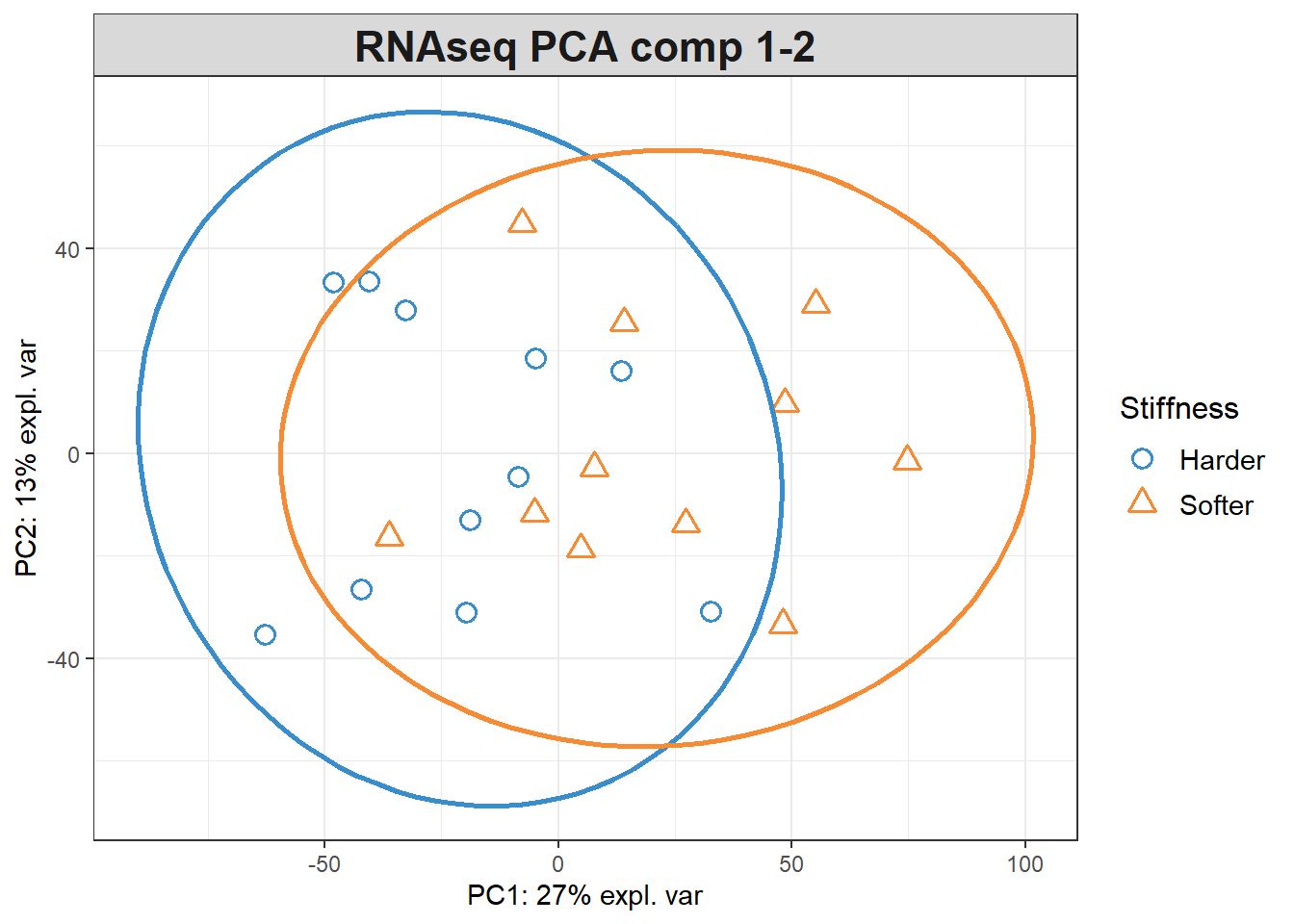

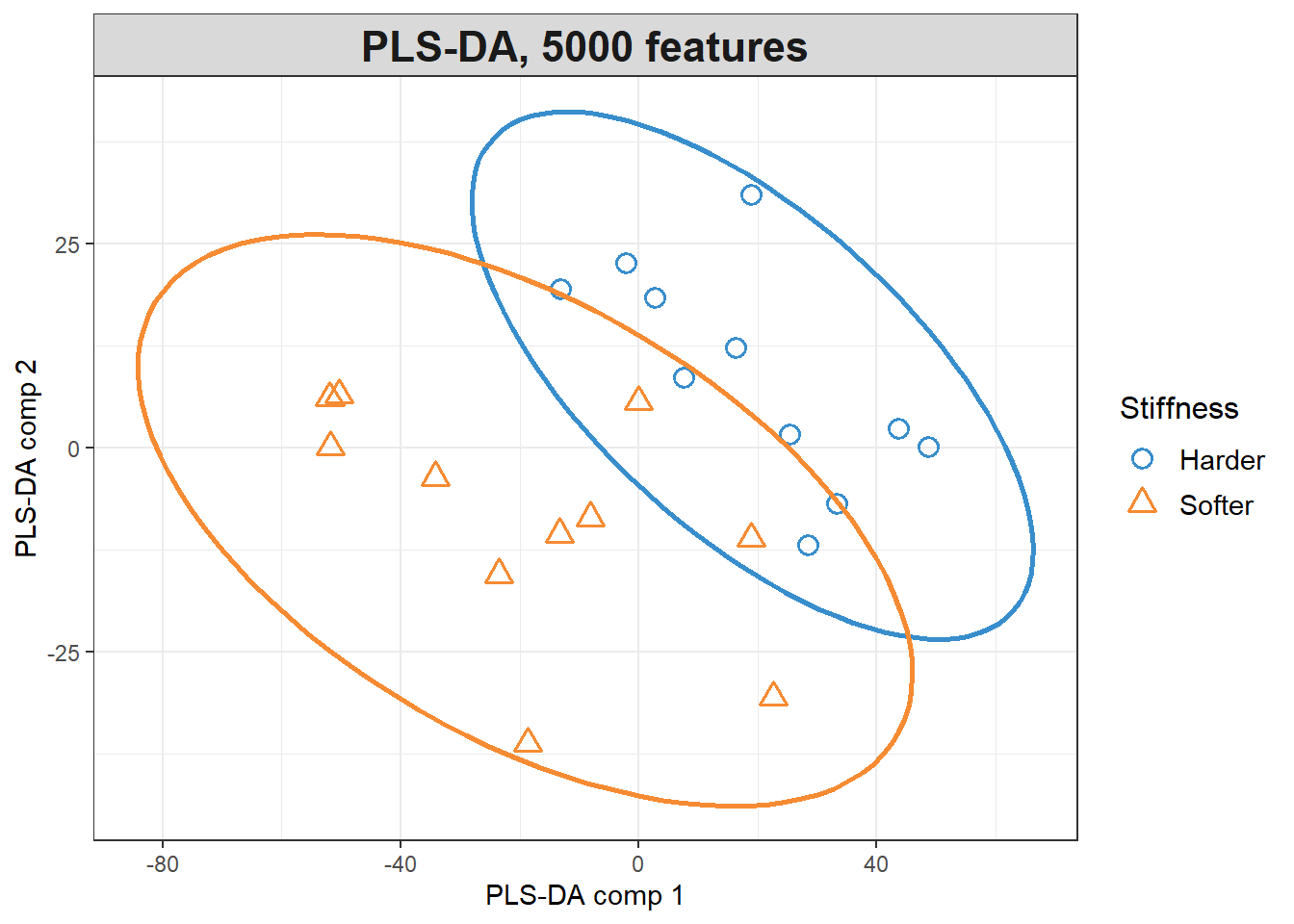

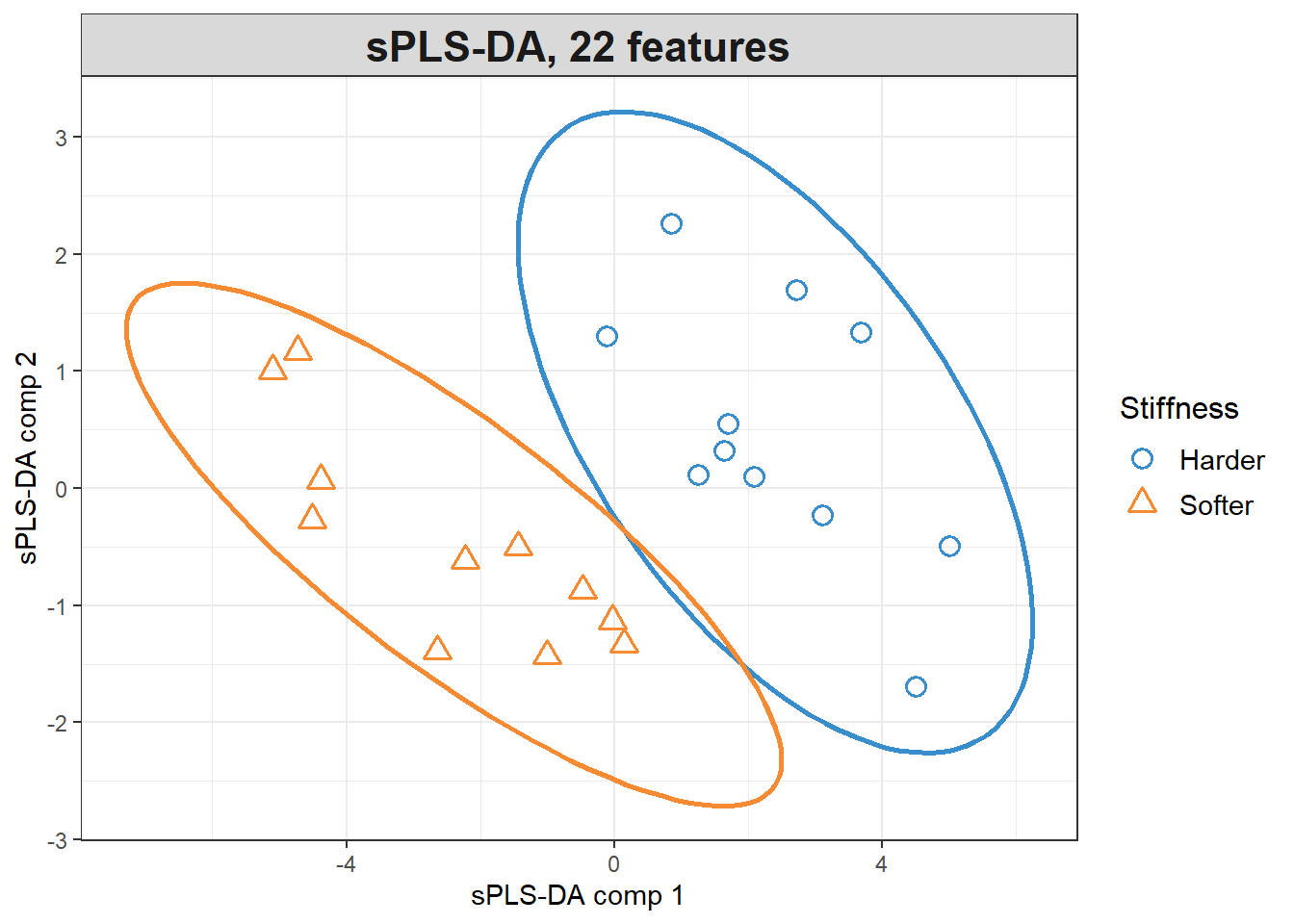


***Supplementary Figure 2:* *Clustering of glioblastoma biopsies based on |G*|_norm_.***  *Biopsies from each patient were classified as ‘stiff’ (blue circles) or ’soft’ (orange triangles) based on the mean stiffness of all biopsies from the same patient. A) Multilevel principal component analysis, accounting for patient variability, shows weak separation of ‘stiff’ and ‘soft’ biopsies along the first principal component. B) Partial least squares differential analysis (PLS-DA) based on the 5000 RNA transcripts with highest variance in the data shows that an expression signal can be found along the first component that distinguishes ‘stiff’ biopsies from ‘soft’ biopsies. C) The tuning of the PLS-DA results revealed that a set of 20 genes along the first component and 2 genes along the second component was sufficient to completely separate the ‘stiff’ and ‘soft’ biopsies.*


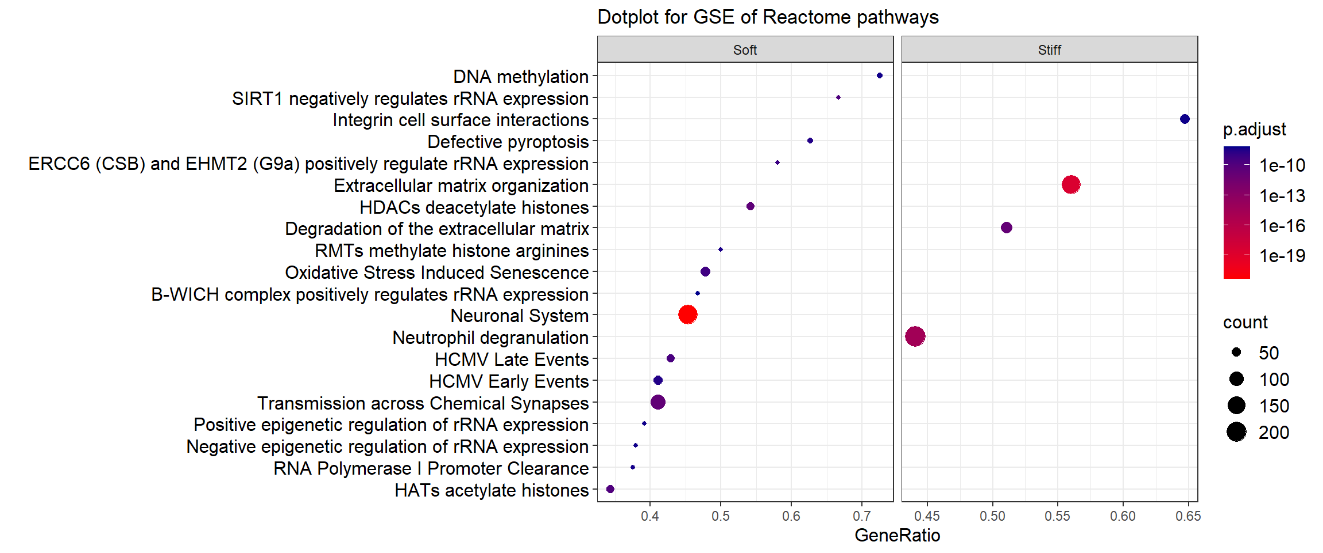

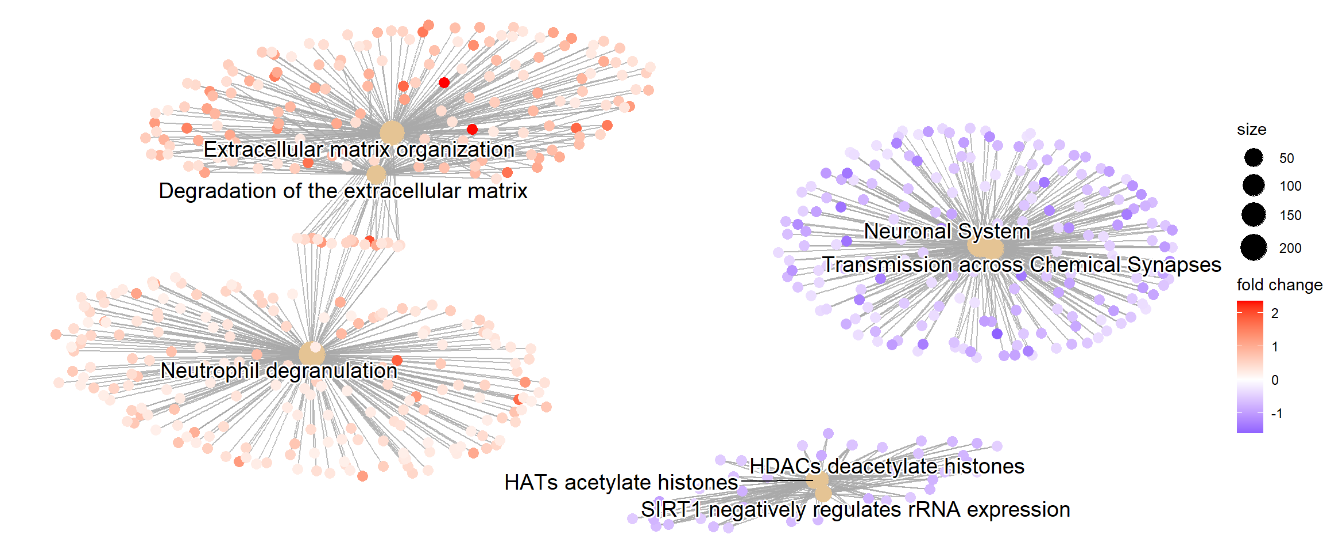


***Supplementary Figure 3. Gene-set enrichment analysis (Reactome pathways) of differentially expressed genes in ‘soft’ and ‘stiff’ tumor biopsies.*** *A) Dotplot representing the terms most highly enriched in Reactome pathways, p.adjust = p-value adjusted for multiple testing. B) Pathway enrichment map of the GSEA results. Central nodes represent Reactome pathways, colored dots represent differentially expressed genes (red = upregulated in ‘stiff’ biopsies, blue = upregulated in ‘soft’ biopsies).*


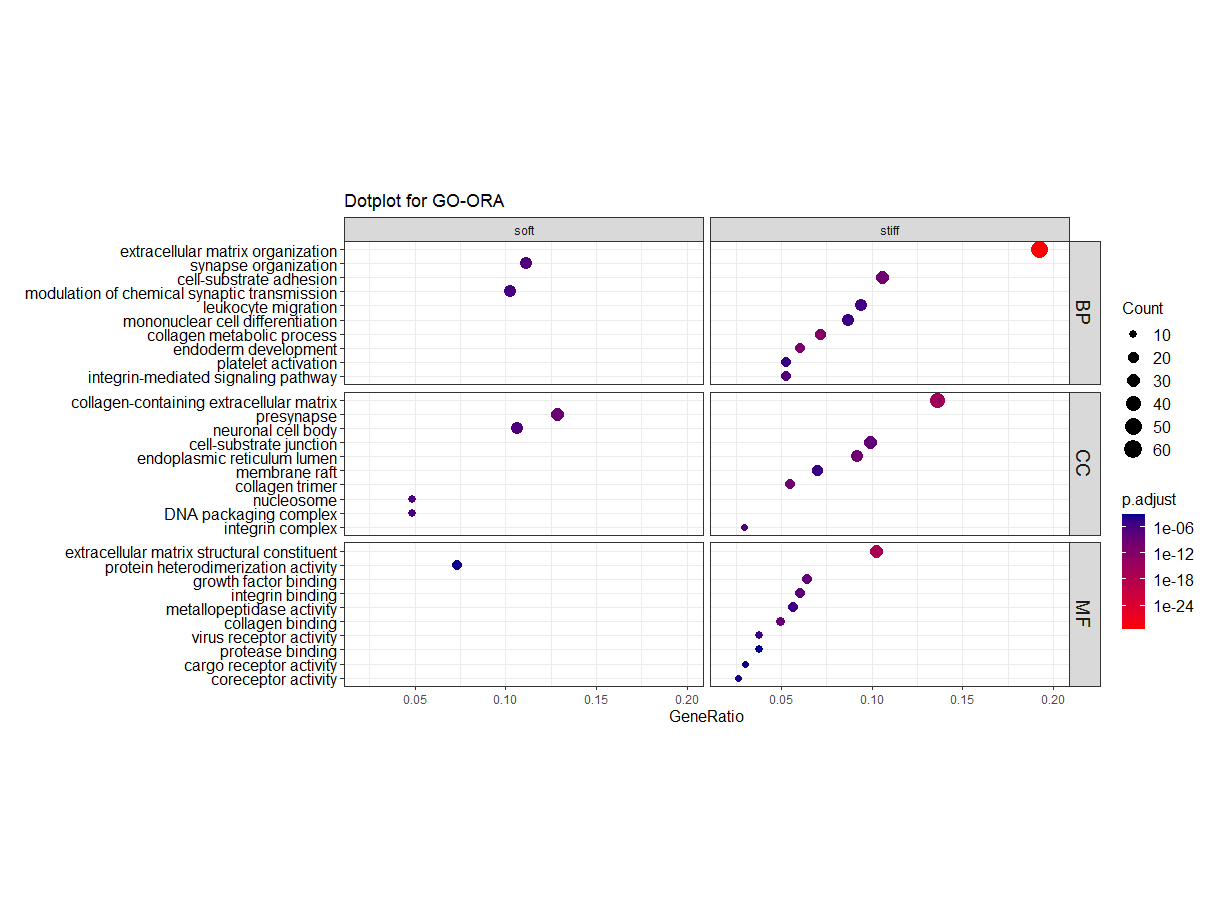


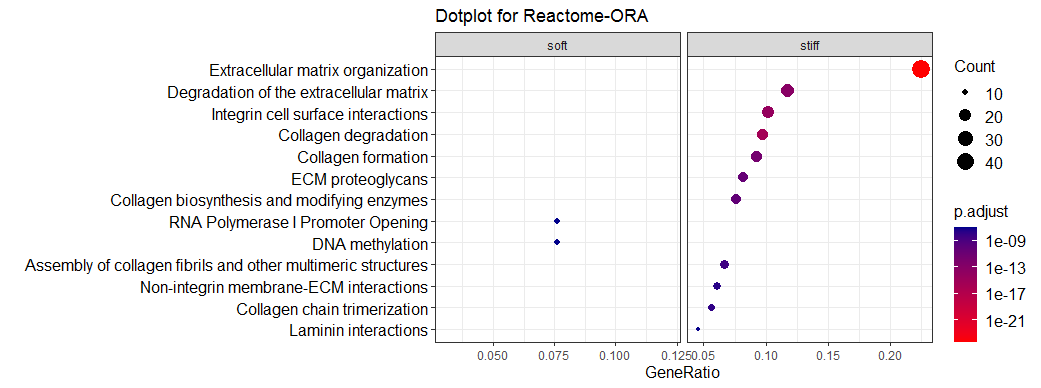


***Supplementary Figure 4:* *GO and Reactome Over-representation analysis.*** *A) Top 10 GO gene sets in each category (BP = biological process, CC = Cellular component, MF = Molecular function) with lowest adjusted p-value in ‘stiff’ and ‘soft’ biopsies.* *B) Top 13 Reactome pathways with lowest adjusted p-value in ‘stiff’ and ’soft’ biopsies.*

| **Patient age** | **Patient gender** | **Tumor location** | **Volume CE tumor [cm^3^]** | **Volume necrosis [cm^3^]** | **Volume FLAIR signal [cm^3^]** | **Mean \|G*\|_norm_ CE tumor** | **Mean \|G*\|_norm_ necrosis** | **Mean \|G*\|_norm_ FLAIR signal** | **Mean** φ**_norm_ CE tumor** | **Mean** φ**_norm_ necrosis** | **Mean** φ**_norm_ FLAIR signal** |
| --- | --- | --- | --- | --- | --- | --- | --- | --- | --- | --- | --- |
| 45-49 | Male | Parietal | 7 | 4 | 51 | 0.80 | 0.66 | 0.99 | 0.99 | 1.01 | 0.95 |
| 75-79 | Male | Temporal | 21 | 12 | 40 | 0.78 | 0.48 | 1.08 | 0.75 | 0.79 | 0.91 |
| 60-64 | Female | Basal ganglia | 29 | 13 | 43 | 0.73 | 0.69 | 0.83 | 0.82 | 0.97 | 0.83 |
| 55-59 | Male | Temporal | 28 | 11 | 162 | 0.80 | 0.64 | 0.89 | 0.79 | 0.67 | 0.82 |
| 35-39 | Female | Parietal | 14 | 4 | 92 | 0.74 | 0.76 | 1.05 | 0.71 | 0.54 | 0.80 |
| 60-64 | Male | Temporal | 28 | 19 | 39 | 0.90 | 0.98 | 1.01 | 0.81 | 0.71 | 0.83 |
| 50-54 | Male | Temporal | 7 | 3 | 45 | 0.84 | 0.65 | 1.21 | 0.97 | 0.98 | 0.80 |
| 50-54 | Female | Temporal | 49 | 25 | 61 | 0.80 | 0.80 | 0.95 | 0.97 | 0.92 | 0.86 |
| 40-44 | Female | Temporal | 5 | 2 | 50 | 0.81 | 0.85 | 1.09 | 0.75 | 0.71 | 0.93 |
| 65-69 | Female | Parietal | 11 | 8 | 2 | 0.98 | 0.77 | 1.21 | 0.88 | 0.82 | 0.85 |
| 65-69 | Female | Parieto-occipital | 39 | 10 | 42 | 0.93 | 0.70 | 0.99 | 0.88 | 1.00 | 0.95 |
| 60-64 | Female | Parieto-occipital | 33 | 45 | 35 | 0.81 | 0.75 | 0.94 | 0.78 | 1.26 | 0.87 |
| 45-49 | Female | Parietal | 13 | 13 | 52 | 0.61 | 0.56 | 0.92 | 0.76 | 0.97 | 0.76 |

**Supplementary Table 1: *Patient characteristics.*** *Patient age group, tumor location, volume, and MRE measurements (normalized to each patient’s contralateral normal-appearing white matter*) *in contrast-enhancing tumor, necrosis, and region with high signal on T2-FLAIR. The first eight patients were used in RNA sequencing analysis.*

| **Covariate** | | | **Univariate analysis** | | | | | **Multivariate analysis** | | | | | |
| --- | --- | --- | --- | --- | --- | --- | --- | --- | --- | --- | --- | --- | --- |
|  |  |  | **B** | **P-value** | **Hazard ratio** | **95% CI for HR** | | **B** | **P-value** | **Hazard ratio** | **95% CI for HR** | |  |
|  |  |  |  |  |  | **Lower** | **Upper** |  |  |  | **Lower** | **Upper** |  |
|  | Gender | 1=female 2=male | 0.043 | 0.771 | 1.043 | 0.783 | 1.39 | 0.046 | 0.756 | 1.047 | 0.785 | 1.396 |  |
|  | Age |  | 0.025 | <.001 | 1.025 | 1.014 | 1.037 | 0.024 | <.001 | 1.024 | 1.013 | 1.036 |  |
| Treatment type | NA | 1 |  | 0.065 |  |  |  |  | 0.068 |  |  |  |  |
|  | Pharmaceutical Therapy, NOS | 2 | 0.324 | 0.08 | 1.383 | 0.963 | 1.986 | 0.356 | 0.054 | 1.427 | 0.994 | 2.05 |  |
|  | Radiation Therapy, NOS | 3 | 0.369 | 0.027 | 1.446 | 1.043 | 2.004 | 0.349 | 0.038 | 1.417 | 1.02 | 1.969 |  |
|  | ECM signature | 1=’Soft’ 2=’Stiff’ | 0.366 | 0.029 | 1.442 | 1.039 | 2.003 | 0.371 | 0.027 | 1.45 | 1.043 | 2.015 |  |

***Supplementary Table 2: Multivariate Cox regression analysis.*** *Univariate and multivariate Cox regression analysis of survival data in the external datasets. Covariates of the model were gender, age, treatment type and expression of genes associated with ‘stiff’ biopsies. Abbreviations: NOS: “not otherwise specified”, B: coefficient of each covariate, CI: confidence interval, HR: hazard ratio*

*Age was found significant in both the univariate and the multivariate analysis. The tumor stiffness gene signature was found to predict a 45 % higher risk of death at any given time. The type of treatment administered to the patients in the external cohorts is also significant, but as the baseline treatment is “NA”, meaning there no treatment information available, we are cautious with interpreting the effect of treatment on survival. However, this is not the focus in our paper, rather to show that gene expression associated with ‘stiff’ biopsies is associated with shorter survival times in this independent dataset, also after adjusting for other covariates.*

| **ENREZ_ID** | **Symbol** | **baseMean** | **log2Fold Change** | **lfcSE** | **P-value** | **Adju. P-value** |
| --- | --- | --- | --- | --- | --- | --- |
| 10351 | ABCA8 | 1204.883261 | 0.671257 | 0.114681 | 4.82E-09 | 7.70E-05 |
| 23213 | SULF1 | 2151.787754 | 1.595155 | 0.319137 | 5.78E-07 | 0.003052 |
| 54885 | TBC1D8B | 118.289721 | 0.950754 | 0.190976 | 6.41E-07 | 0.003052 |
| 4121 | MAN1A1 | 903.7229709 | 0.890446 | 0.180097 | 7.64E-07 | 0.003052 |
| 79839 | CCDC102B | 1032.172753 | 0.922098 | 0.188653 | 1.02E-06 | 0.003258 |
| 3875 | KRT18 | 62.60869999 | 1.415651 | 0.294371 | 1.52E-06 | 0.003951 |
| 8829 | NRP1 | 4671.780854 | 0.982309 | 0.205396 | 1.73E-06 | 0.003951 |
| 5325 | PLAGL1 | 113.3530688 | 0.996035 | 0.209931 | 2.09E-06 | 0.004172 |
| 5592 | PRKG1 | 463.8615734 | 0.792318 | 0.172652 | 4.45E-06 | 0.007111 |
| 81551 | STMN4 | 407.1583395 | -0.97506 | 0.211744 | 4.13E-06 | 0.007111 |
| 2803 | GOLGA4 | 4826.145609 | 0.274702 | 0.060761 | 6.15E-06 | 0.008938 |
| 767 | CA8 | 198.8150717 | -0.9019 | 0.201271 | 7.43E-06 | 0.009888 |
| 1909 | EDNRA | 900.7642216 | 0.760681 | 0.17067 | 8.31E-06 | 0.010156 |
| 78997 | GDAP1L1 | 109.6847843 | -1.2734 | 0.286655 | 8.90E-06 | 0.010156 |
| 5175 | PECAM1 | 829.4028375 | 0.52938 | 0.12002 | 1.03E-05 | 0.010502 |
| 115548 | FCHO2 | 1475.189333 | 0.419033 | 0.0951 | 1.05E-05 | 0.010502 |
| 1290 | COL5A2 | 8963.612758 | 0.747587 | 0.170538 | 1.17E-05 | 0.010964 |
| 5205 | ATP8B1 | 252.8700371 | 0.809535 | 0.185212 | 1.24E-05 | 0.010983 |
| 7373 | COL14A1 | 860.8016235 | 0.945786 | 0.217792 | 1.41E-05 | 0.011838 |
| 9332 | CD163 | 12876.52315 | 1.308098 | 0.306163 | 1.93E-05 | 0.013013 |
| 9169 | SCAF11 | 4011.135905 | 0.300244 | 0.069986 | 1.79E-05 | 0.013013 |
| 875 | CBS | 961.7247316 | -0.49534 | 0.11545 | 1.78E-05 | 0.013013 |
| 93145 | OLFM2 | 808.9923179 | -0.58671 | 0.137404 | 1.96E-05 | 0.013013 |
| 441381 | LRRC24 | 33.97029124 | -0.90986 | 0.212358 | 1.83E-05 | 0.013013 |
| 3759 | KCNJ2 | 563.1353637 | 0.537025 | 0.126575 | 2.21E-05 | 0.013239 |
| 23216 | TBC1D1 | 1671.086313 | 0.291718 | 0.068805 | 2.24E-05 | 0.013239 |
| 345630 | FBLL1 | 33.27600773 | -1.23017 | 0.289728 | 2.18E-05 | 0.013239 |
| 26136 | TES | 368.73219 | 0.691061 | 0.16345 | 2.36E-05 | 0.013454 |
| 79187 | FSD1 | 176.8332367 | -0.60704 | 0.14422 | 2.56E-05 | 0.014123 |
| 4124 | MAN2A1 | 2722.209833 | 0.500494 | 0.119305 | 2.73E-05 | 0.014524 |
| 22925 | PLA2R1 | 289.4342541 | 0.796937 | 0.190545 | 2.88E-05 | 0.014864 |
| 1.01E+08 | DNM3OS | 135.1473547 | 1.177743 | 0.283332 | 3.23E-05 | 0.015651 |
| 84910 | TMEM87B | 790.3001554 | 0.373412 | 0.089841 | 3.23E-05 | 0.015651 |
| 5139 | PDE3A | 650.0727524 | 0.808889 | 0.195392 | 3.48E-05 | 0.016329 |
| 1378 | CR1 | 428.468695 | 1.721665 | 0.417006 | 3.65E-05 | 0.016655 |
| 84935 | MEDAG | 187.4729426 | 1.736515 | 0.422237 | 3.91E-05 | 0.016886 |
| 1601 | DAB2 | 1367.228764 | 0.89499 | 0.217557 | 3.89E-05 | 0.016886 |
| 3672 | ITGA1 | 3155.083673 | 0.892766 | 0.218458 | 4.38E-05 | 0.01705 |
| 55075 | UACA | 2801.043162 | 0.879256 | 0.215 | 4.32E-05 | 0.01705 |
| 4253 | MIA2 | 751.9145183 | 0.190349 | 0.046427 | 4.13E-05 | 0.01705 |
| 128312 | H2BU1 | 56.06297646 | -0.94549 | 0.231187 | 4.32E-05 | 0.01705 |
| 10184 | LHFPL2 | 2846.391379 | 0.668107 | 0.1639 | 4.58E-05 | 0.017402 |
| 57026 | PDXP | 301.3936541 | -0.66022 | 0.162233 | 4.71E-05 | 0.017496 |
| 1600 | DAB1 | 56.07243773 | -0.74573 | 0.183607 | 4.87E-05 | 0.017697 |
| 4060 | LUM | 1454.232745 | 1.585698 | 0.394083 | 5.73E-05 | 0.0182 |
| 728264 | CARMN | 714.9461116 | 1.138235 | 0.284377 | 6.27E-05 | 0.0182 |
| 1282 | COL4A1 | 25705.42138 | 0.927032 | 0.229689 | 5.44E-05 | 0.0182 |
| 1396 | CRIP1 | 139.1756583 | 0.882014 | 0.219741 | 5.97E-05 | 0.0182 |
| 3676 | ITGA4 | 1081.835497 | 0.598904 | 0.149273 | 6.02E-05 | 0.0182 |
| 29803 | REPIN1 | 985.3827012 | -0.28253 | 0.070259 | 5.79E-05 | 0.0182 |
| 728875 | NA | 71.47935093 | -0.54967 | 0.135915 | 5.25E-05 | 0.0182 |
| 1468 | SLC25A10 | 69.37581705 | -0.70006 | 0.174792 | 6.20E-05 | 0.0182 |
| 1152 | CKB | 2415.056651 | -0.88914 | 0.220577 | 5.55E-05 | 0.0182 |
| 494470 | RNF165 | 193.396958 | -0.94091 | 0.232927 | 5.36E-05 | 0.0182 |
| 11075 | STMN2 | 428.509595 | -1.54268 | 0.385229 | 6.21E-05 | 0.0182 |
| 9568 | GABBR2 | 317.4056257 | -1.42335 | 0.356861 | 6.65E-05 | 0.018965 |
| 4327 | MMP19 | 498.6702733 | 1.640407 | 0.416131 | 8.08E-05 | 0.020003 |
| 9509 | ADAMTS2 | 357.1242342 | 1.535422 | 0.389542 | 8.09E-05 | 0.020003 |
| 948 | CD36 | 401.8675628 | 1.213807 | 0.306493 | 7.49E-05 | 0.020003 |
| 9358 | ITGBL1 | 219.7353646 | 1.084708 | 0.274643 | 7.83E-05 | 0.020003 |
| 219623 | TMEM26 | 150.0464163 | 0.815724 | 0.206018 | 7.51E-05 | 0.020003 |
| 81792 | ADAMTS12 | 312.2400418 | 0.801566 | 0.202922 | 7.81E-05 | 0.020003 |
| 285203 | EOGT | 659.0670372 | 0.484049 | 0.12306 | 8.37E-05 | 0.020003 |
| 359845 | RFLNB | 399.7972356 | 0.467286 | 0.118812 | 8.39E-05 | 0.020003 |
| 7067 | THRA | 1744.471032 | -0.57394 | 0.145132 | 7.67E-05 | 0.020003 |
| 283248 | RCOR2 | 193.5926837 | -1.0851 | 0.275763 | 8.32E-05 | 0.020003 |
| 375704 | ENHO | 168.7048087 | -1.41831 | 0.360117 | 8.20E-05 | 0.020003 |
| 23317 | DNAJC13 | 3629.359927 | 0.181268 | 0.046274 | 8.96E-05 | 0.020776 |
| 90268 | OTULIN | 1024.722629 | 0.164369 | 0.041965 | 8.97E-05 | 0.020776 |
| 7855 | FZD5 | 453.0463433 | 0.510575 | 0.130528 | 9.17E-05 | 0.020922 |
| 140738 | TMEM37 | 115.1899765 | 0.92 | 0.23587 | 9.60E-05 | 0.021364 |
| 1496 | CTNNA2 | 2181.699365 | -0.4864 | 0.124725 | 9.63E-05 | 0.021364 |
| 1803 | DPP4 | 393.6808038 | 1.46781 | 0.377505 | 0.000101 | 0.022 |
| 51310 | SLC22A17 | 1001.60094 | -0.55445 | 0.142678 | 0.000102 | 0.022 |
| 57524 | CASKIN1 | 89.75823766 | -0.90651 | 0.233705 | 0.000105 | 0.02235 |
| 3559 | IL2RA | 322.7840705 | 1.407741 | 0.364006 | 0.00011 | 0.022532 |
| 1295 | COL8A1 | 785.7219972 | 0.829723 | 0.214528 | 0.00011 | 0.022532 |
| 7048 | TGFBR2 | 1865.961745 | 0.557067 | 0.143881 | 0.000108 | 0.022532 |
| 1303 | COL12A1 | 2159.00124 | 1.090289 | 0.283196 | 0.000118 | 0.023709 |
| 2335 | FN1 | 90865.15825 | 1.020001 | 0.265024 | 0.000119 | 0.023709 |
| 7476 | WNT7A | 60.23711675 | -0.64025 | 0.166501 | 0.00012 | 0.023743 |
| 23075 | SWAP70 | 1735.632773 | 0.261018 | 0.068001 | 0.000124 | 0.02412 |
| 4320 | MMP11 | 136.6725256 | 0.979662 | 0.255476 | 0.000126 | 0.024199 |
| 6362 | CCL18 | 165.8221877 | 3.206418 | 0.8398 | 0.000134 | 0.024614 |
| 3487 | IGFBP4 | 1330.361348 | 1.005899 | 0.264911 | 0.000146 | 0.024614 |
| 9060 | PAPSS2 | 534.3162319 | 0.717231 | 0.188686 | 0.000144 | 0.024614 |
| 4162 | MCAM | 3578.341451 | 0.554437 | 0.145774 | 0.000143 | 0.024614 |
| 2321 | FLT1 | 3181.634308 | 0.525557 | 0.137997 | 0.00014 | 0.024614 |
| 4094 | MAF | 1185.587343 | 0.470618 | 0.123125 | 0.000132 | 0.024614 |
| 9208 | LRRFIP1 | 1882.434969 | 0.334148 | 0.087816 | 0.000142 | 0.024614 |
| 5311 | PKD2 | 1730.840704 | 0.267787 | 0.070224 | 0.000137 | 0.024614 |
| 4597 | MVD | 198.203436 | -0.53868 | 0.141385 | 0.000139 | 0.024614 |
| 4915 | NTRK2 | 10188.32693 | -0.65318 | 0.171955 | 0.000146 | 0.024614 |
| 26232 | FBXO2 | 188.0271482 | -0.90256 | 0.237649 | 0.000146 | 0.024614 |
| 51617 | NSG2 | 330.3962473 | -1.48427 | 0.38894 | 0.000136 | 0.024614 |
| 4625 | MYH7 | 116.0433084 | -0.7667 | 0.202208 | 0.00015 | 0.024902 |
| 1278 | COL1A2 | 10955.85651 | 1.338037 | 0.354165 | 0.000158 | 0.025246 |
| 54829 | ASPN | 244.8656243 | 1.23206 | 0.325763 | 0.000156 | 0.025246 |
| 57125 | PLXDC1 | 1016.990503 | 0.824465 | 0.218261 | 0.000158 | 0.025246 |
| 154141 | MBOAT1 | 132.9346344 | 0.808017 | 0.21415 | 0.000161 | 0.025246 |
| 30061 | SLC40A1 | 1744.7296 | 0.43417 | 0.115026 | 0.00016 | 0.025246 |
| 138311 | DIPK1B | 300.7735757 | -0.46717 | 0.123724 | 0.000159 | 0.025246 |
| 6781 | STC1 | 751.7672591 | 1.197282 | 0.317934 | 0.000166 | 0.025498 |
| 54796 | BNC2 | 387.8907464 | 0.787387 | 0.208994 | 0.000165 | 0.025498 |
| 4026 | LPP | 7269.585995 | 0.49995 | 0.132952 | 0.00017 | 0.025568 |
| 6538 | SLC6A11 | 255.5257214 | -1.00746 | 0.267829 | 0.000169 | 0.025568 |
| 8338 | H2AC20 | 610.1125553 | -0.58486 | 0.155735 | 0.000173 | 0.025828 |
| 2444 | FRK | 45.02494024 | 0.807139 | 0.215587 | 0.000181 | 0.02634 |
| 2200 | FBN1 | 3836.740463 | 0.689311 | 0.184011 | 0.00018 | 0.02634 |
| 10924 | SMPDL3A | 308.1054271 | 0.670435 | 0.179163 | 0.000183 | 0.02634 |
| 9697 | TRAM2 | 777.0399409 | 0.634394 | 0.169563 | 0.000183 | 0.02634 |
| 8654 | PDE5A | 931.6264987 | 0.644951 | 0.172872 | 0.000191 | 0.02706 |
| 51560 | RAB6B | 1252.662182 | -0.53887 | 0.144465 | 0.000191 | 0.02706 |
| 55803 | ADAP2 | 953.2199966 | 0.61838 | 0.165884 | 0.000193 | 0.027066 |
| 203522 | INTS6L | 432.7831097 | 0.268365 | 0.072046 | 0.000195 | 0.027139 |
| 5551 | PRF1 | 194.6917989 | 1.233593 | 0.331646 | 0.0002 | 0.027242 |
| 4643 | MYO1E | 1517.086364 | 0.580884 | 0.156161 | 0.000199 | 0.027242 |
| 3912 | LAMB1 | 6558.519009 | 0.952069 | 0.256399 | 0.000205 | 0.027469 |
| 27 | ABL2 | 2714.176905 | 0.308347 | 0.08303 | 0.000204 | 0.027469 |
| 11326 | VSIG4 | 3914.818203 | 1.06168 | 0.286626 | 0.000212 | 0.02801 |
| 10522 | DEAF1 | 529.1343407 | -0.28902 | 0.078005 | 0.000211 | 0.02801 |
| 144402 | CPNE8 | 334.4890902 | 0.631622 | 0.170661 | 0.000215 | 0.028114 |
| 23175 | LPIN1 | 1576.370062 | -0.27185 | 0.073812 | 0.000231 | 0.029701 |
| 1826 | DSCAM | 836.6862168 | -0.89297 | 0.242412 | 0.00023 | 0.029701 |
| 121457 | IKBIP | 710.8423048 | 0.456273 | 0.124168 | 0.000238 | 0.03044 |
| 5300 | PIN1 | 505.7290614 | -0.2893 | 0.078845 | 0.000243 | 0.030466 |
| 23580 | CDC42EP4 | 2054.20444 | -0.5124 | 0.139757 | 0.000246 | 0.030466 |
| 844 | CASQ1 | 102.851126 | -0.62236 | 0.169543 | 0.000242 | 0.030466 |
| 55964 | SEPTIN3 | 919.5956037 | -0.87965 | 0.239917 | 0.000246 | 0.030466 |
| 677828 | SNORA47 | 176.1461107 | -0.49527 | 0.135231 | 0.00025 | 0.030707 |
| 221395 | ADGRF5 | 1868.792441 | 0.592567 | 0.162029 | 0.000255 | 0.030861 |
| 59342 | SCPEP1 | 1196.295538 | 0.478 | 0.130639 | 0.000253 | 0.030861 |
| 57089 | ENTPD7 | 391.9679173 | 0.532667 | 0.145848 | 0.00026 | 0.031226 |
| 8685 | MARCO | 535.2883966 | 2.576484 | 0.70937 | 0.000281 | 0.031385 |
| 114904 | C1QTNF6 | 189.8862482 | 0.889486 | 0.245128 | 0.000285 | 0.031385 |
| 1368 | CPM | 886.7084237 | 0.739726 | 0.203536 | 0.000279 | 0.031385 |
| 11214 | AKAP13 | 6303.945295 | 0.338091 | 0.092877 | 0.000272 | 0.031385 |
| 10921 | RNPS1 | 1012.036306 | -0.16959 | 0.046521 | 0.000267 | 0.031385 |
| 51222 | ZNF219 | 186.2433482 | -0.36032 | 0.099032 | 0.000274 | 0.031385 |
| 79007 | DBNDD1 | 248.8072732 | -0.39024 | 0.107414 | 0.00028 | 0.031385 |
| 3757 | KCNH2 | 289.7502657 | -0.5773 | 0.158908 | 0.00028 | 0.031385 |
| 23542 | MAPK8IP2 | 253.6324494 | -0.69246 | 0.190819 | 0.000285 | 0.031385 |
| 9363 | RAB33A | 76.79579553 | -0.86168 | 0.236206 | 0.000264 | 0.031385 |
| 162494 | RHBDL3 | 397.04649 | -0.95185 | 0.261879 | 0.000278 | 0.031385 |
| 10690 | FUT9 | 1111.737231 | -1.03623 | 0.28554 | 0.000285 | 0.031385 |
| 51696 | HECA | 1206.177572 | 0.254234 | 0.070139 | 0.000289 | 0.031558 |
| 374875 | HSD11B1L | 155.8397076 | -0.35897 | 0.09906 | 0.00029 | 0.031558 |
| 84892 | POMGNT2 | 534.3183284 | -0.39647 | 0.109508 | 0.000294 | 0.031737 |
| 5799 | PTPRN2 | 575.3596982 | -0.46404 | 0.129048 | 0.000323 | 0.034659 |
| 132720 | FAM241A | 76.4253114 | 0.88035 | 0.245329 | 0.000333 | 0.034731 |
| 22795 | NID2 | 874.5732207 | 0.822026 | 0.228902 | 0.000329 | 0.034731 |
| 9695 | EDEM1 | 1378.143463 | 0.459338 | 0.12798 | 0.000332 | 0.034731 |
| 155185 | AMZ1 | 90.55429743 | -0.70038 | 0.195173 | 0.000333 | 0.034731 |
| 4017 | LOXL2 | 1457.428712 | 0.995069 | 0.277577 | 0.000337 | 0.034986 |
| 51393 | TRPV2 | 314.0000926 | 0.668317 | 0.186517 | 0.000339 | 0.034987 |
| 1374 | CPT1A | 1281.279583 | 0.492052 | 0.137443 | 0.000344 | 0.035179 |
| 23040 | MYT1L | 219.6461449 | -1.58061 | 0.441958 | 0.000348 | 0.035445 |
| 7402 | UTRN | 8581.245903 | 0.315511 | 0.088689 | 0.000374 | 0.037852 |
| 1284 | COL4A2 | 11792.98347 | 0.860112 | 0.242032 | 0.00038 | 0.038159 |
| 2261 | FGFR3 | 971.5925916 | -0.73788 | 0.207961 | 0.000388 | 0.038734 |
| 10052 | GJC1 | 1509.788124 | 0.453422 | 0.127883 | 0.000392 | 0.038853 |
| 57084 | SLC17A6 | 62.45771606 | -2.08441 | 0.588143 | 0.000394 | 0.038853 |
| 1E+08 | WHAMMP1 | 332.1194823 | 0.488077 | 0.138238 | 0.000414 | 0.040369 |
| 4713 | NDUFB7 | 564.652786 | -0.42705 | 0.120918 | 0.000413 | 0.040369 |
| 4312 | MMP1 | 333.0243407 | 2.357464 | 0.669073 | 0.000426 | 0.041023 |
| 10912 | GADD45G | 122.9919302 | -1.2734 | 0.361557 | 0.000428 | 0.041023 |
| 114794 | ELFN2 | 170.5098342 | -1.57895 | 0.448354 | 0.000429 | 0.041023 |
| 4642 | MYO1D | 416.4996328 | 0.805727 | 0.23039 | 0.00047 | 0.043232 |
| 1075 | CTSC | 3371.493764 | 0.737876 | 0.210894 | 0.000467 | 0.043232 |
| 55614 | KIF16B | 690.9023115 | 0.338261 | 0.096565 | 0.00046 | 0.043232 |
| 5978 | REST | 1979.939898 | 0.336081 | 0.096128 | 0.000472 | 0.043232 |
| 23607 | CD2AP | 1148.566558 | 0.310308 | 0.088542 | 0.000457 | 0.043232 |
| 130612 | TMEM198 | 185.5749796 | -0.45957 | 0.131483 | 0.000474 | 0.043232 |
| 56967 | C14orf132 | 2304.611287 | -0.55489 | 0.158338 | 0.000458 | 0.043232 |
| 53826 | FXYD6 | 1795.861564 | -0.776 | 0.222009 | 0.000473 | 0.043232 |
| 8600 | TNFSF11 | 17.21627915 | 1.563785 | 0.448565 | 0.00049 | 0.043657 |
| 3101 | HK3 | 469.1411728 | 1.060946 | 0.303903 | 0.000481 | 0.043657 |
| 133584 | EGFLAM | 198.9539668 | 1.056084 | 0.303028 | 0.000492 | 0.043657 |
| 8038 | ADAM12 | 1172.340227 | 0.752182 | 0.2158 | 0.000491 | 0.043657 |
| 23086 | EXPH5 | 272.076768 | -0.593 | 0.170044 | 0.000488 | 0.043657 |
| 5069 | PAPPA | 487.7788881 | 1.45492 | 0.417961 | 0.0005 | 0.043837 |
| 1306 | COL15A1 | 500.8326701 | 1.293265 | 0.371434 | 0.000498 | 0.043837 |
| 4323 | MMP14 | 4320.572043 | 0.607981 | 0.174775 | 0.000504 | 0.043837 |
| 3688 | ITGB1 | 11064.35875 | 0.540154 | 0.155421 | 0.00051 | 0.043837 |
| 29965 | CDIP1 | 575.7181599 | -0.3303 | 0.095043 | 0.00051 | 0.043837 |
| 55228 | PNMA8A | 1059.662381 | -0.52364 | 0.150668 | 0.00051 | 0.043837 |
| 23640 | HSPBP1 | 306.095839 | -0.28365 | 0.081887 | 0.000532 | 0.04548 |
| 79812 | MMRN2 | 390.28342 | 0.577056 | 0.166768 | 0.00054 | 0.045616 |
| 83539 | CHST9 | 431.0490011 | -0.60842 | 0.175791 | 0.000538 | 0.045616 |
| 10882 | C1QL1 | 281.9994608 | -0.775 | 0.224379 | 0.000552 | 0.04644 |
| 9890 | PLPPR4 | 779.0493132 | -0.6626 | 0.192343 | 0.000571 | 0.047567 |
| 2066 | ERBB4 | 707.39527 | -0.98605 | 0.286254 | 0.000572 | 0.047567 |
| 1824 | DSC2 | 431.3027964 | 0.806546 | 0.234836 | 0.000594 | 0.049132 |
| 284716 | RIMKLA | 240.5064459 | -0.5165 | 0.150504 | 0.0006 | 0.049369 |
| 9211 | LGI1 | 126.2127386 | -0.86347 | 0.251749 | 0.000604 | 0.049465 |
| 55702 | YJU2 | 270.6322614 | -0.26324 | 0.076798 | 0.000609 | 0.049608 |

***Supplementary Table 3: Differential expression results****. Differential gene expression between ‘stiff’ and ‘soft’ biopsies in 8 glioblastoma patients (22 biopsies). Only genes with an adjusted p-value (Benjamini and Hochberg method) below 0.05 are shown. Genes with higher expression in ‘stiff’ biopsies have log2foldchange>0, genes with higher expression in ‘soft’ biopsies have log2foldchange<0.*
